# The Naturally Bioactive Vicine Extracted from Faba Beans Is Responsible for the Transformation of Grass Carp (*Ctenopharyngodon idella*) into Crisp Grass Carp

**DOI:** 10.3390/antiox14070813

**Published:** 2025-07-01

**Authors:** Xinyu Zheng, Minyi Luo, Bing Fu, Gen Kaneko, Jingjing Tian, Jun Xie, Jilun Hou, Ermeng Yu

**Affiliations:** 1 Key Laboratory of Tropical and Subtropical Fishery Resources Application and Cultivation, Ministry of Agriculture and Rural Affairs, Pearl River Fisheries Research Institute Chinese Academy of Fishery Sciences, Guangzhou 510380, China; tianjj@prfri.ac.cn (J.T.); xiejunhy01@126.com (J.X.); 2 State Key Laboratory of Mariculture Biobreeding and Sustainable Goods, Beidaihe Central Experiment Station, Chinese Academy of Fishery Sciences, Qinhuangdao 066100, China; zhengxy@bces.ac.cn; 3 Hebei Key Laboratory of the Bohai Sea Fish Germplasm Resources Conservation and Utilization, Beidaihe Central Experiment Station, Chinese Academy of Fishery Sciences, Qinhuangdao 066100, China; 4 Bohai Sea Fishery Research Center, Chinese Academy of Fishery Science, Qinhuangdao 066100, China; 5 Guangxi Academy of Marine Sciences, Guangxi Academy of Sciences, Nanning 530000, China; 6 College of Natural and Applied Science, University of Houston-Victoria, Victoria, TX 77901, USA; kanekog@uhv.edu; 7 College of Marine Sciences, South China Agricultural University, Guangzhou 510640, China; fub@stu.scau.edu.cn; 8 Agricultural Service Center of Xiaolan Town, Zhongshan 528415, China; 15914605449@163.com

**Keywords:** reactive oxygen species, oxidant stress, vicine, grass carp, textural quality

## Abstract

While faba bean feeding improves grass carp muscle texture via reactive oxygen species (ROS), the main bioactive compound was unclear. In this study, vicine—a pro-oxidant glycoside—was isolated from faba beans using cation-exchange column chromatography and supplemented into the feed of grass carp at 0.6%. To assess the impact of vicine on muscle texture, the grass carp were fed for 150 days with three treatments: control group, faba bean group, and vicine group. The results showed that vicine improved muscle texture similarly to faba beans but caused fewer adverse effects on muscle, liver, and intestinal health. Vicine improved grass carp muscle texture in the following ways: (1) induced ROS overproduction, activating the Caspase apoptosis pathway and downregulating *Pax-7* to promote satellite cell-mediated myofiber regeneration; (2) vicine-mediated intestinal microbiota alterations increased lipopolysaccharide (LPS) levels, indirectly elevating muscle ROS via the gut–muscle axis to further affect muscle structure. This study demonstrated that vicine improved muscle texture by activating ROS-dependent myofiber regeneration but also induced oxidative stress and gut microbiota perturbation. While vicine mitigated the severe toxicity of faba beans, its application requires careful evaluation of its toxicological properties to balance benefits and risks. This study offers new insights for enhancing the quality of aquatic animals.

## 1. Introduction

The sensory quality of aquatic products, particularly muscle texture, is a pivotal determinant of consumer preference. Crisp grass carp (*Ctenopharyngodon idella*), a commercially important cultivar developed through long-term faba bean (*Vicia faba*) feeding, has gained substantial popularity due to its improved muscle texture [1,2]. In 2022, the industrial output value of crisp grass carp in Zhongshan, Guangdong Province, exceeded 1.5 billion RMB (approximately 2.05 × 10^8^ USD), with products not only dominating regional markets in Guangdong but also being exported to North America and Southeast Asia [3]. Beyond grass carp, faba bean feeding has improved muscle texture in several aquaculture species, including tilapia (*Oreochromis niloticus*) and Yellow River carp (*Cyprinus carpio*), thus underscoring the broad applicability of this dietary strategy [4,5].

While faba bean feeding effectively enhances the muscle texture of fish, prolonged administration induces adverse effects such as hemolysis, hepatopancreatic injury, intestinal inflammation, and metabolic disorders [6,7,8]. Previous studies using water and ethanol extracts of faba beans demonstrated a promising solution: these extracts achieved the muscle-texture benefits of faba beans while reducing their detrimental side effects in grass carp and tilapia [1,4,9]. Mechanistic investigations linked faba bean-induced muscle texture changes in grass carp to ROS generation [9,10]; however, the specific bioactive compound in faba beans responsible for triggering ROS-mediated myofiber remodeling remains uncharacterized.

Among the anti-nutritional factors in faba beans—including tannins, phytic acid, saponins, and vicine—our focus is on vicine due to its unique oxidative potential [11]. Previous studies have established that tannins, phytic acid, and saponins primarily impact fish intestinal health and immune function [12,13,14], with no reported association with muscle texture modification. Vicine (2,6-diamino-4,5-dihydroxypyrimidine-5-β-D-glucopyranoside, C_10_H_16_N_4_O_7_), a glycosidic compound abundant in faba beans (Gardiner, Marquardt, Kemp, 1988; Pulkkinen, 2015) [15,16], is metabolized by intestinal microbial β-glucosidase to form divicine (2,6-diamino-4,5-dihydroxypyrimidine, C_4_H_6_N_4_O_2_) [17]. In humans, divicine triggers ROS generation in erythrocytes, depleting glutathione (GSH) and nicotinamide adenine dinucleotide phosphate (NADPH) to induce favism in individuals with glucose-6-phosphate dehydrogenase (G6PD) deficiency [18,19]. This metabolic pathway inspired our hypothesis that vicine mediates the beneficial effects of faba beans on fish muscle texture, supported by three key lines of evidence: (1) omic analysis of “crispy” grass carp linked the muscle texture improvement to ROS [2]; (2) vicine’s high oxidative capacity was confirmed, and antioxidant supplementation abolished faba bean-induced muscle texture improvement [10,17]; (3) fish fed diets containing faba beans or faba bean extracts exhibited oxidative stress symptoms analogous to human favism [1]. In summary, we hypothesize that vicine is the key bioactive molecule in faba beans responsible for improving grass carp muscle texture via ROS-mediated mechanisms.

Current research on the extraction and isolation of vicine from faba beans remains limited. Most existing methods are confined to quantitative analysis or small-scale purification of vicine, typically requiring expensive equipment, substantial time, and large volumes of organic reagents. For instance, the method employed by Jamalian et al. necessitates a high concentration of trichloroacetic acid [20]; Mahmoud et al.’s protocol takes up to four days to complete faba bean pretreatment [21]; and Marquardt et al.’s approach requires extensive acetone usage and three days of static separation [22]. Previous studies have shown that vicine exhibits strong polarity [23,24], making it suitable for separation and extraction via ion exchange chromatography. The 001×7 resin is widely utilized in high-purity water preparation and bioactive substance extraction due to its cost-effectiveness [25]. In this study, 001×7 cation exchange resin was employed to adsorb and separate vicine from faba bean water extract, thereby reducing organic reagent consumption and time costs during the extraction process.

In this study, we first developed a simple and efficient method for the extraction and purification of vicine from faba beans, using a combination of water extraction with cation exchange column chromatography. Subsequently, a 150-day feeding trial was conducted using three dietary treatments: compound feed (control group), faba bean-supplemented feed (faba bean group), and compound feed supplemented with purified vicine (vicine group). Evaluated parameters included hematological indexes; muscle texture; muscle microstructure; and ROS levels in muscle, hepatopancreas, and intestine; as well as intestinal microbiota composition. Quantitative real-time PCR (qRT-PCR) was performed to quantify the gene expression related to oxidative–antioxidant pathways, apoptosis signaling, muscle proliferation/differentiation pathways, and myofibrillar protein synthesis in grass carp muscle tissue. These analyses aimed to determine whether vicine serves as the key bioactive component driving muscle texture changes in grass carp.

## 2. Materials and Methods

### 2.1. Animal Ethics Statement

The experimental design and procedures of the present research have been approved by the Animal Ethics Committee of the Pearl River Fisheries Research Institute, Chinese Academy of Fishery Sciences (CAFS). The approval number is: LAEC-PRFRI-2023-02-03.

### 2.2. Extraction and Purification of Vicine

Faba bean aqueous extracts were prepared following the protocol reported in a previous study [1]. In brief, faba bean powder was homogenized with distilled water at a 1:12 (*w*/*v*) ratio. The pH of the mixture was adjusted to 9.0 using 1 M NaOH or HCl, followed by temperature equilibration to 45 °C. The suspension was then subjected to ultrasonic treatment at 40 kHz for 1 h. After sonication, the mixture was filtered through a 200-mesh sieve to remove insoluble materials and the supernatant was collected. The pH of the supernatant was adjusted to 4.0 by adding 4 M HCL, and the solution was allowed to stand at room temperature for 12 h to promote precipitation. Following centrifugation (5000× *g* for 10 min), the supernatant was transferred to a rotary evaporator and concentrated to dryness under reduced pressure at 55 °C, yielding the faba bean water extract.

The faba bean water extract was dissolved in 5 volumes (*w*/*v*) of distilled water, and the pH of the solution was adjusted to 3.0. Subsequently, the solution was loaded onto a 001×7 cation exchange resin column (Ningbo ZhengGuang Co., Ltd., Ningbo, China) and subjected to cation exchange column chromatography at a flow rate of 1.5 bed volume (BV) per hour. The column was washed with 1.0 BV of water, and vicine was eluted using a 0.5% ammonia solution. The eluent was then concentrated and further purified with acetone. After that, the pH of the solution was adjusted to neutral to induce the crystallization of vicine. The obtained vicine crystals were redissolved in water, and the pH of the resulting solution was adjusted to neutral, which was then left undisturbed for recrystallization.

Three grams (3.0 g) of faba bean powder were precisely weighed and mixed with 12 mL of distilled water. The pH of the mixture was adjusted to 9.0, followed by ultrasonic extraction at 45 °C for 1 h. The extract was centrifuged at 5000 rpm for 10 min at room temperature, and the supernatant was collected. To the remaining bean residue, 6 mL of distilled water was added, and the extraction procedure was repeated three additional times, resulting in a total of four extractions. The pH of each combined extract was adjusted to 4.5–5.0 with hydrochloric acid to induce protein precipitation, which was then refrigerated at 4 °C overnight. The samples were centrifuged at 8000 rpm for 10 min at 4 °C to separate the supernatant from the precipitated proteins. The vicine content in the four supernatants was quantified by high-performance liquid chromatography (HPLC), and the total vicine content in the faba bean powder was calculated as the sum of the four extracted amounts.

### 2.3. High Performance Liquid Chromatography (HPLC) Analysis of Vicine

Vicine standard (purity ≥ 98%) was purchased from Chengdu Herbsubstance Co., Ltd. (Chengdu, Sichuan, China). The purity of the extracted vicine was analyzed using a Shimadzu LC20AT high-performance liquid chromatography (HPLC) system (Shimadzu, Kyoto, Japan) equipped with an ECOSIL 120-5-C18 AQ column (250 mm × 4.6 mm, 5 μm). The mobile phase consisted of 0.3% formic acid aqueous solution, delivered at a flow rate of 0.8 mL/min. The detection wavelength was set at 275 nm, the column temperature was maintained at 30 °C, and the injection volume was 10 μL. Prior to analysis, all samples were filtered through a 0.22 μm polymeric membrane filter. Vicine in the samples was identified by comparing retention times with the standard compound and co-injection experiments.

Quantitative analysis was performed using a standard curve (Y = 64.132X, *R*^2^ = 0.9998) constructed with standard vicine solutions at concentrations of 4, 8, 15, 25, 50, and 100 μg/mL. The standard curve was corrected by subtracting the blank mobile phase to eliminate background interference. Additionally, spike recovery experiments (n = 6) were conducted, demonstrating a quantitative error < 2%, which met the methodological requirements for accuracy and reliability.

### 2.4. Preparation of Vicine Feed

**Figure 1:**
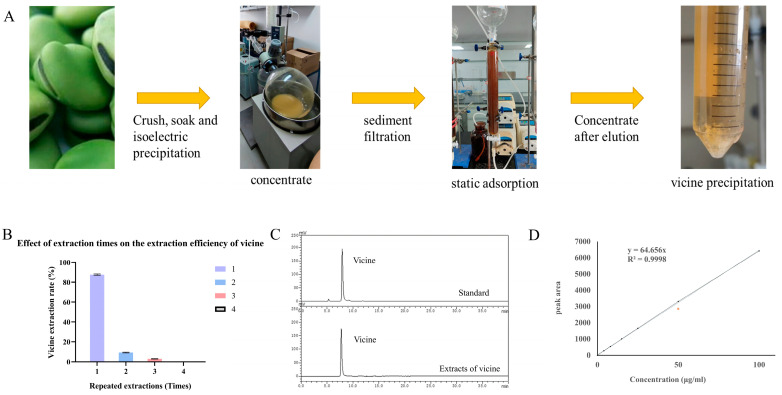
Extraction and purity verification of vicine. (**A**) Schematic overview of the extraction and purification processes. (**B**) Extraction yields of vicine across sequential extractions. (**C**) HPLC chromatograms of vicine samples and standard compounds. (**D**) Quantification of vicine purity using a calibration curve (The orange dots represent the quantified purity of vicine after the first purification, which reached 86.4%.).

The formulations of the three experimental diets are shown in Table 1. Control group: Based on the conventional formula for grass carp compound feed. Faba bean group: 90% of the control diet components were replaced with natural faba bean powder to simulate the traditional feeding method for crispy grass carp, with 10% other raw materials added to balance the nutrient composition. The vicine content in natural faba bean powder was determined by HPLC as 0.60 ± 0.02% (n = 3 batches, Figure S1), resulting in a theoretical vicine concentration of 900 g/kg × 0.6% = 5.4 g/kg (0.54%, *w*/*w*) in the diet. Vicine group: Laboratory-purified vicine (purity 98.5%, prepared by cation exchange column chromatography and recrystallization, Figure 1D) was added to the control diet at a level of 6 g/kg (0.6%, *w*/*w*), designed to mimic the effects of individual ingredients at naturally occurring exposure levels. Diets were processed into 3 mm diameter strips using an electric meat grinder (Yongkang Taibao Electric Appliance, Yongkang, China), air-dried at room temperature, and cut into 3 mm long pellets. High temperatures were strictly avoided during production to prevent thermal degradation of vicine. Crude protein, crude fat, calcium, moisture, and crude fiber contents of the diets were determined with reference to previous studies, according to Chinese National Standard methods respectively [9].

### 2.5. Feeding Trial

The 150-day feeding trial was conducted at the Pearl River Fisheries Research Institute. Grass carp (initial body weight: 180 ± 10 g) were obtained from Zhongshan Food and Aquatic Products Import and Export Group Co., Ltd. (Zhongshan, China) and stocked in nine cement pools (2 m × 2 m × 1 m, 12 fish per pool). The experimental design included three groups—control, faba bean, and vicine. Fish were fed twice daily at 09:00 and 16:00, with the daily feeding rate set at 2% of their body weight. Water quality parameters were maintained consistently across all groups: pH 7.5–8.0, dissolved oxygen ≥7.0 mg/L, and water temperature 25–30 °C. For each treatment group, three independent culture ponds (n = 3) were used as biological replicates. Three fish were randomly selected from each pond for measurement, and the mean value of each pond was calculated as an independent data point for statistical analysis (i.e., n = 3 ponds per group, with n = 3 technical replicates per pond).

### 2.6. Sampling Procedures

Following the 150-day culture period, three grass carp individuals were randomly sampled from each pool and euthanized using pH-buffered tricaine methanesulfonate (MS-222, 250 mg/L). Body weight, body length, visceral mass, hepatopancreas mass, and abdominal fat mass were measured to calculate growth-related parameters as follows:

Weight gain rate (WGR, %) = (final weight − initial weight)/initial weight × 100.

Condition factor (CF, %) = body weight/length^3^ × 100.

Hepatopancreas somatic index (HSI, %) = hepatopancreas weight/body weight × 100.

Visceral somatic index (VSI, %) = visceral weight/body weight × 100.

Abdominal fat index (AFI, %) = abdominal fat weight/body weight × 100.

Feed conversion rate (FCR, %) = total food intake/(final weight − initial weight).

Survival rate (SR, %) = terminal survival mantissa/initial mantissa × 100%.

Prior to euthanasia, blood samples were collected from the caudal vein of grass carp. Muscles adjacent to the lateral line were excised for texture profile analysis (TPA), hematoxylin and eosin (H&E) staining, collagen content determination, and other subsequent assays. Following complete removal of the intestinal contents, a 5 mm segment of the midgut was fixed in Bouin’s reagent for 18 h for H&E staining and histopathological evaluation. A 2 mm^3^ tissue block from the hepatopancreas was similarly fixed in Bouin’s reagent for 18 h and stained with oil red O for lipid deposition analysis. Additional hepatopancreas samples were collected and stored at −80 °C for further biochemical detection. All tissue samples were processed and stored under standardized conditions to ensure analytical consistency.

### 2.7. Measurement of Textural Quality Parameters and Collagen Content

Texture profile analysis (TPA) of grass carp dorsal muscle was conducted using a Universal TA Texture Analyzer (Shanghai Tengba, Shanghai, China). The evaluated parameters were hardness, gumminess, springiness, chewiness, cohesiveness, and resilience, as described in previous studies [1,26]. Muscle samples (2.0 cm × 2.0 cm × 1.0 cm) were excised from the lateral line region and analyzed with a cylindrical probe (P35, diameter 35 mm). Pre-test, test, and post-test speeds were set at 2 mm/s, 1 mm/s, and 5 mm/s, respectively. The interval between two compressions was 2 s, and the compression ratio was 25%. Collagen content in muscle tissue was quantified using an Ultra-Sensitive Fish Collagen ELISA Kit (Sino Biological, Beijing, China; Kit No. YX-E21992F).

### 2.8. Histological Analysis

The H&E staining of muscle and midgut tissues, as well as oil red O staining of the hepatopancreas, were performed following standard histology protocols. Muscle fiber characteristics, including diameter and density, were quantified by counting fibers in the field of view using DP2-BSW 2.2 software (Build 6212; Olympus, Tokyo, Japan), as described in a previous study [2]. For oil red O-stained hepatopancreas sections, the relative area ratio of lipid droplets was analyzed using Image-Pro Plus 6.0 (Media Cybernetics, Rockville, MD, USA) [27]. Intestinal morphological parameters—including lamina propria (LP) thickness, eosinophilic granulocyte (EG) density, mucosal fold (MF) height, and muscularis externa (MC) thickness—were quantitatively evaluated from three tissue slides per fish.

### 2.9. Tissue Biochemical Analysis

Grass carp tissues were analyzed with ultra-sensitive fish ELISA kits from Sino Best Biological Technology Co., Ltd. (Shanghai, China): total ROS (T-ROS, YX-E22123F), hydrogen peroxide (H_2_O_2_, YX-E21807F), hydroxyl radicals (·OH, YX-E22083F), malondialdehyde (MDA, JM-07937F1), superoxide dismutase (SOD, YX-E22111F), glutathione peroxidase (GSH, YX-E22086F), catalase (CAT, YX-E22107F), superoxide anion (SOA, YX-E22109F), oxygen free radicals (OFR, YX-E22077F), cytochrome C (Cyto-C, YX-E22061F), mitochondrial membrane permeability pore (MPTP, YX-E22087F), complex I (COX I, YX-E22093F), complex III (COX III, YX-E22094F), complex IV (COX IV, YX-E22095F), nicotinamide adenine dinucleotide phosphate (NADPH, YX-E22101F), glucose-6-phosphate dehydrogenase (G6PD, YX-E22078F), acid phosphatase (ACP, JM-07753F1), alkaline phosphatase (AKP, JM-07758F1), and lipopolysaccharide (LPS, JM-03762F1). Alanine aminotransferase (AST), aspartate aminotransferase (ALT), triglyceride (TG), total cholesterol (TC), high-density lipoprotein-C (HDL-C), and low density lipoprotein-C (LDL-C) were detected by automatic biochemical analyzer (Mindray, Shenzhen, China).

### 2.10. Quantitative Real-Time PCR

Three fish per replicate pond (n = 3 fish/pond, n = 9 fish/group) from three ponds per treatment group were sampled. Approximately 0.5 g dorsal muscle tissue was dissected from each fish, immediately frozen in liquid nitrogen, and stored at −80 °C. Samples from three fish in each pond were pooled as one biological replicate (n = 3 biological replicates/group). Total RNA was extracted using TRIzol reagent (Invitrogen, Cat. No. 15596026, Shanghai, China) following the manufacturer’s protocol. RNA purity and integrity were verified by agarose gel electrophoresis, and its concentration was quantified using an Implen NanoPhotometer (Implen Inc., Munich, Germany). First-strand cDNA was synthesized from 1 μg of total RNA using the Takara Reverse Transcription Kit (Takara, Shiga, Japan, Cat. No. 2690A) following the manufacturer’s instructions. Quantitative real-time PCR (qRT-PCR) was performed on a LightCycler^®^ 96 Real-Time PCR System (Roche, Basel, Switzerland) using 20 μL reaction mixtures containing 0.8 μL of each primer (10 μM), 6.4 μL nuclease-free water, 2 μL diluted cDNA template, and 10 μL 2× SYBR Premix Ex Taq II (TaKaRa, Japan, Cat. No. RR390A). The thermal cycling protocol was as follows: initial denaturation at 95 °C for 5 min, followed by 45 cycles of 95 °C for 15 s and 60 °C for 1 min. Negative controls included no-cDNA templates and DNase-treated non-reverse-transcribed RNA samples to monitor contamination. Primer sequences are listed in Table S1, with β-actin serving as the housekeeping gene. Relative gene expression was calculated using Pfaffl’s method, normalizing to the housekeeping gene and calibrating against a reference sample.

### 2.11. Intestinal Microbial Sequencing and Analysis

Microbial DNA was extracted from intestinal samples using the Zymo Research BIOMICS DNA Microprep Kit (D4301, Orange, CA, USA). DNA integrity was evaluated via 0.8% agarose gel electrophoresis, and nucleic acid concentration was quantified using a Tecan F200 microplate reader with the PicoGreen dye method. The V4 hypervariable region of the 16S rRNA gene was amplified using the primers 515F (5′-GTGYCAGCMGCCGCGGTAA-3′) and 806R (5′-GGACTACHVGGGTWTCTAAT-3′). PCR reactions were performed under the following conditions: initial denaturation at 94 °C for 1 min; 25–30 cycles of 94 °C for 20 s (denaturation), 54 °C for 30 s (annealing), and 72 °C for 30 s (extension); a final extension at 72 °C for 5 min; and storage at 4 °C. Each sample was amplified in triplicate, and equimolar amounts of PCR products were pooled for subsequent library preparation.

PCR products were mixed with 6× loading buffers and separated by 2% agarose gel electrophoresis to visualize target fragments (≈400 bp). The Zymoclean Gel Recovery Kit (D4008, Zymo Research) was used to extract qualified amplicons, which were then quantified using a Qubit^®^ 2.0 Fluorometer (Thermo Scientific, Waltham, MA, USA). Equal-molar libraries were prepared and sequenced on an Illumina HiSeq platform using the HiSeq Rapid SBS Kit v2 (500 Cycle, FC-402-4023) with paired-end 250 bp (PE250) sequencing.

Double-end reads were merged using FLASH and quality-filtered in QIIME 2. Sequence noise and chimeras were removed via the Deblur algorithm to generate amplicon sequence variants (ASVs). Taxonomic classification of ASVs was performed using a Naïve Bayes classifier trained on the SILVA 138 database. Community composition was analyzed at the phylum, order, family, genus, and species levels. Alpha diversity indices (Chao1, Shannon, Simpson, and Good’s coverage) were calculated in R, and linear discriminant analysis effect size (LEfSe) was applied to identify differentially abundant biomarkers with an LDA score ≥ 3.

### 2.12. Statistical Analyses

Statistical analyses were conducted using IBM SPSS Statistics 22 (SPSS Inc., Chicago, IL, USA). Data were first subjected to one-way analysis of variance (ANOVA), followed by Duncan’s multiple range test to evaluate significant differences among the experimental groups. A significance threshold of *p* < 0.05 was applied to determine statistical significance. Results are reported as means ± standard deviations (SDs).

## 3. Results

### 3.1. Extraction, Purification, and Identification of Vicine

The overall workflow for vicine extraction and purification is illustrated in Figure 1A. Sequential extraction yields were 87.7 ± 0.7%, 9.1 ± 0.4%, and 3.0 ± 0.3% for the first, second, and third extractions, respectively (Figure 1B). HPLC analysis of the combined extracts revealed a single, distinct peak corresponding to vicine (Figure 1C), confirming successful isolation. No vicine was detected in the fourth extraction, indicating complete extraction within the first three cycles. Notably, two consecutive extractions achieved a total vicine extraction efficiency of 97.8 ± 1.3%, demonstrating the method’s high recovery capacity. The faba bean seeds used in this study contained 0.60 ± 0.02% vicine (*w*/*w*).

A standard curve constructed with vicine solutions (4.0–100.0 μg/mL) enabled quantification of the purified vicine, which exhibited a concentration of 50 mg/mL and a purity of 86.4% (Figure 1D). Following recrystallization, the purity of vicine was further enhanced to 98.5%, meeting the requirements for subsequent biological assays.

### 3.2. Growth Performance and Muscle Texture, Biochemistry and Microstructure of Grass Carp After Feeding on Vicine

As shown in Table 2, the control group exhibited significantly higher weight gain rate (WGR), condition factor (CF), and feed conversion ratio (FCR) than both the faba bean and vicine groups (*p* < 0.05). The vicine group displayed a significantly greater WGR than the faba bean group (*p* < 0.05), indicating superior growth performance with pure vicine supplementation. Conversely, the hepatic somatic index (HSI) and abdominal fat index (AFI) were notably higher in the faba bean and vicine groups compared to the control group. Among these, the vicine group showed a significantly higher AFI than the faba bean group (*p* < 0.05), suggesting a stronger effect of pure vicine on abdominal fat accumulation. Survival rate (SR) differed significantly across groups: the faba bean group had a lower SR than both the control and vicine groups (*p* < 0.05), whereas the control and vicine groups maintained 100% survival throughout the trial.

**Figure 2:**
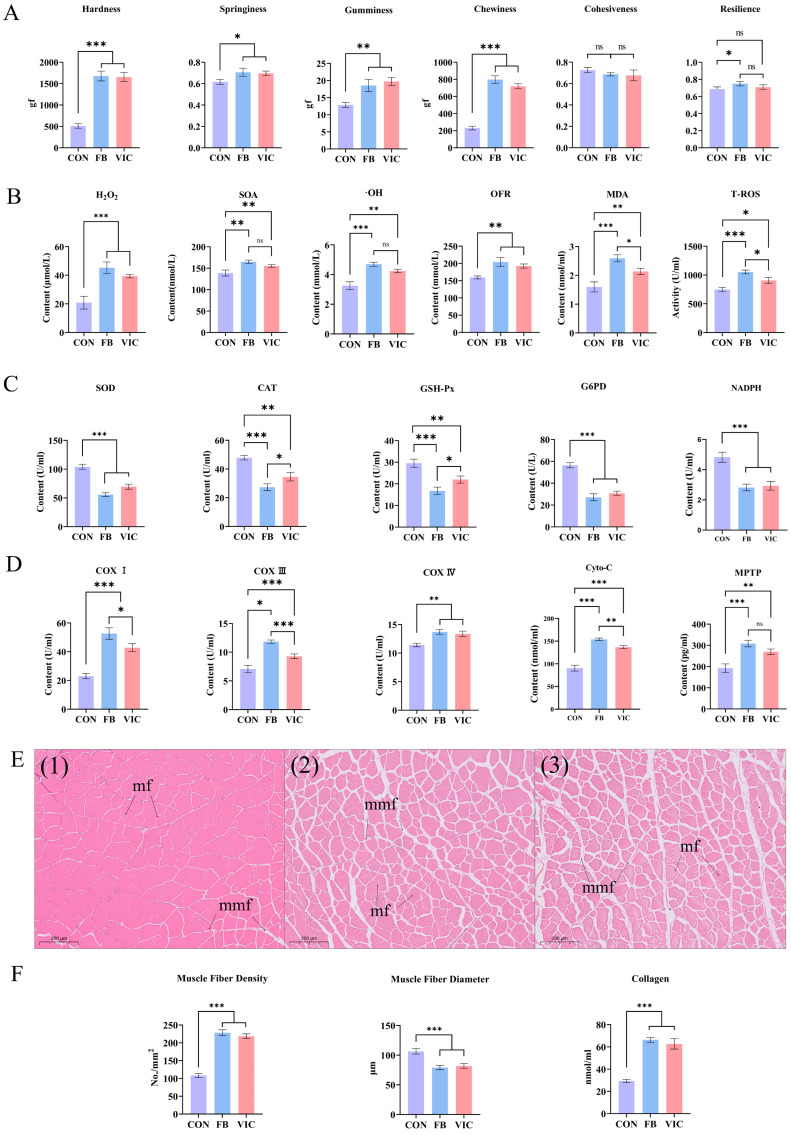
Texture, biochemical parameters, and microstructural characteristics of grass carp muscle following vicine ingestion. CON: control group; FB: faba bean group; VIC: vicine group. (**A**) TPA parameters: hardness, springiness, gumminess, chewiness, cohesiveness, and resilience. (**B**) Quantification of ROS and oxidative products: H_2_O_2_, SOA, ·OH, OFR, MDA, and T-ROS. (**C**) Antioxidant enzyme activities and redox molecules: SOD, GSH-Px, CAT, NADPH, and G6PD. (**D**) Mitochondrial electron transport chain components: cytochrome c oxidase complexes (COXI, COXIII, COXIV), Cyto-C, and MPTP. (**E**) Transverse sections of grass carp muscle: (1) CON, (2) FB, (3) VIC (scale bar = 200 μm, H&E staining). mf: muscle fiber; mmf: muscle matrix fiber. (**F**) Muscle fiber characteristics (diameter, number density) and collagen content. For each treatment group, three ponds were used (three fish per pond), and data are presented as the mean ± SD of the ponds. Statistical significance was evaluated using one-way ANOVA followed by Duncan’s test, *** *p* < 0.001, ** *p* < 0.01, or * *p* < 0.05.

Compared with the control group, the faba bean and vicine groups exhibited significantly higher muscle hardness, gumminess, springiness, chewiness, cohesiveness, and resilience (Figure 2A, *p* < 0.05). No significant differences were observed between the faba bean and vicine groups in any of these texture parameters (*p* > 0.05), indicating that both treatments equivalently enhanced the textural properties of grass carp muscle. Specifically, supplementation with either faba bean or vicine promoted significant increases in all measured TPA parameters compared to the control (*p* < 0.05), suggesting a consistent effect on improving muscle texture through these interventions.

No significant differences in muscle fiber diameter or number density were observed between the faba bean and vicine groups. However, both treatment groups exhibited significantly higher muscle fiber number density than the control group (Figure 2F, *p* < 0.05), indicating a common effect of faba bean and vicine on promoting muscle fiber proliferation. For muscle collagen content, no significant difference was detected between the faba bean and vicine groups (*p* > 0.05), but both had significantly higher collagen levels than the control group (Figure 2F, *p* < 0.05), suggesting that these treatments similarly enhanced collagen deposition in grass carp muscle.

Compared with the faba bean group, the levels of ROS, including MDA and T-ROS, in the grass carp muscles of the vicine group were significantly reduced. Meanwhile, the activities of antioxidant enzymes, such as GSH-Px, and CAT, showed an upward trend. However, when compared with the control group, the ROS levels in the grass carp muscles of both the faba bean group and the vicine group increased significantly, and the antioxidant enzyme activities decreased significantly (Figure 2B,C, *p* < 0.05).

In the vicine group, the levels of mitochondrial electron transport chain (ETC) components associated with ROS generation—including COXI, COXIII, and COXIV—were significantly lower than those in the faba bean group, except for COXIV, which showed no significant difference (*p* > 0.05). Both the vicine and faba bean groups exhibited significantly higher COXI, COXIII, and COXIV levels than the control group (*p* < 0.05). For cell apoptosis-related markers, Cyto-C and MPTP levels were elevated in both treatment groups compared to the control, with the faba bean group showing a more pronounced increase than the vicine group (Figure 2D, *p* < 0.05). Regarding muscle microstructure, the vicine group displayed significantly higher muscle fiber number density, collagen content, and muscle fiber interstitial area than the faba bean group (Figure 2E, *p* < 0.05). Both the faba bean and vicine groups exhibited upward trends in these parameters compared to the control group (Figure 2F, *p* < 0.05).

### 3.3. Effects of Vicine on Blood Biochemistry of Grass Carp

**Figure 3:**
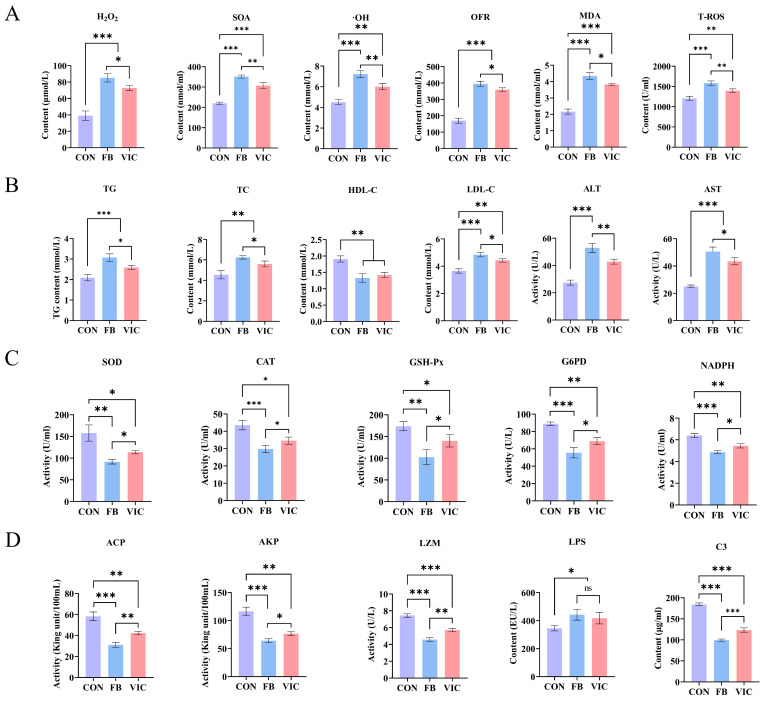
Effects of vicine on blood biochemistry of grass carp. CON: control group; FB: faba bean group; VIC: vicine group. (**A**) Quantification of ROS and oxidative products: H_2_O_2_, SOA, ·OH, OFR, MDA, and T-ROS. (**B**) Blood lipid metabolism and liver function indices: TG, TC, HDL-C, LDL-C, ALT, and AST. (**C**) Antioxidant enzyme activities and redox molecules: SOD, GSH-Px, CAT, NADPH, and G6PD. (**D**) Serum immune-related enzyme levels: ACP, AKP, LSZ, LPS, and C3. For each treatment group, three ponds were used (three fish per pond), and data are presented as the mean ± SD of the ponds. Statistical significance was evaluated using one-way ANOVA followed by Duncan’s test, *** *p* < 0.001, ** *p* < 0.01, or * *p* < 0.05.

In the vicine group, the levels of ROS—H_2_O_2_, SOA, ·OH, OFR, MDA, and tT-ROS—in grass carp blood were significantly lower than those in the faba bean group (*p* < 0.05). However, both the faba bean and vicine groups exhibited higher ROS levels compared to the control group (Figure 3A, *p* < 0.05). Antioxidant enzyme activities, including SOD, GSH-Px, CAT, NADPH, and G6PD, were significantly higher in the vicine group than in the faba bean group (Figure 3C, *p* < 0.05).

Faba bean supplementation significantly increased blood TG, TC, ALT, AST, and LDL-C in grass carp. The vicine group showed a similar trend but with significantly lower levels than the faba bean group (*p* < 0.05), although both treatment groups remained significantly higher than the control group (Figure 3B). HDL-C did not differ significantly between the faba bean and vicine groups, but both were significantly lower than the control group (*p* < 0.05).

Compared with the control group, grass carp fed with faba beans or vicine exhibited significantly reduced serum levels of immune-related enzymes (ACP, AKP, LSZ) and inflammatory factors (LPS, complement C3) (*p* < 0.05, Figure 3D). Specifically, the faba bean group showed significantly lower levels of complement ACP, AKP, C3 and LSZ than the vicine group, whereas LPS levels did not differ significantly between the two treatment groups. (Figure 3D) (*p* < 0.05).

### 3.4. Effects of Vicine on Biochemical Parameters, Microstructure and Microbiota of the Intestine of Grass Carp

**Figure 4:**
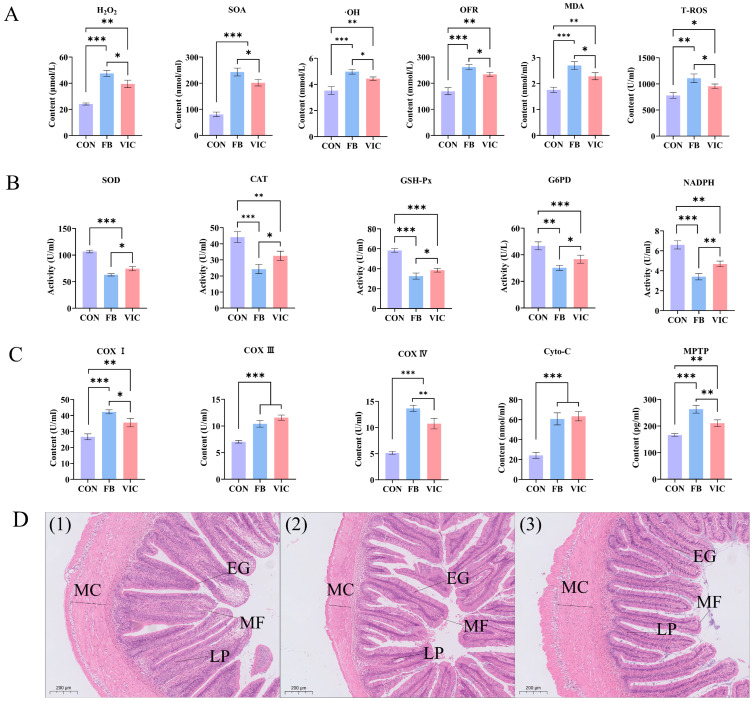
Biochemical and histological analysis of the grass carp intestine. CON: control group; FB: faba bean group; VIC: vicine group. (**A**) Quantification of ROS and oxidative products: H_2_O_2_, SOA, ·OH, OFR, MDA, and T-ROS. (**B**) Antioxidant enzyme activities and redox molecules: SOD, GSH-Px, CAT, NADPH, and G6PD. (**C**) Mitochondrial electron transport chain components: cytochrome c oxidase complexes (COXI, COXIII, COXIV), Cyto-C, and MPTP. (**D**) Intestinal histological analysis: representative micrographs of transverse sections [(1) CON, (2) FB, (3) VIC; H&E staining, scale bar = 200 μm]. LP: lamina propria, EG: eosinophilic granulocyte, MF: mucosal fold, and MC: muscularis externa. For each treatment group, three ponds were used (three fish per pond), and data are presented as the mean ± SD of the ponds. Statistical significance was evaluated using one-way ANOVA followed by Duncan’s test, *** *p* < 0.001, ** *p* < 0.01, or * *p* < 0.05.

Intestinal ROS levels—including H_2_O_2_, SOA, ·OH, OFR, MDA, and T-ROS—were significantly higher in the faba bean group than in the control group (*p* < 0.05, Figure 4A). The vicine group exhibited a similar upward trend compared to the control but had significantly lower H_2_O_2_, SOA, ·OH, OFR, MDA, and T-ROS levels than the faba bean group (*p* < 0.05).

Antioxidant enzyme activities (SOD, GSH-Px, CAT, NADPH, G6PD) in both the faba bean and vicine groups were significantly lower than those in the control group (*p* < 0.05, Figure 4B). However, the vicine group displayed notably higher activities of these antioxidant enzymes compared to the faba bean group, indicating a partial mitigation of oxidative stress by vicine supplementation.

Analysis of mitochondrial ETC components in the grass carp intestine revealed that both faba bean and vicine treatments increased levels of COXI, COXIII, COXIV, Cyto-C, and MPTP compared to the control group (*p* < 0.05, Figure 4C). Notably, the faba bean group exhibited significantly higher COXI, COXIV, and MPTP levels than the vicine group (*p* < 0.05), indicating more pronounced mitochondrial membrane dysfunction and ETC activation in the faba bean treatment.

Histological examination (H&E staining) showed that the faba bean group had sparse mucosal folds, degraded villi, reduced villus height, and thinner intestinal muscularis externa (outer muscle layer) compared to the control. In contrast, the vicine group maintained a villus height similar to the control and displayed thickened intestinal muscularis, with minimal signs of mucosal damage (Figure 4D). These findings collectively suggest that vicine supplementation induced less structural damage to the grass carp intestine than faba bean inclusion.

**Figure 5:**
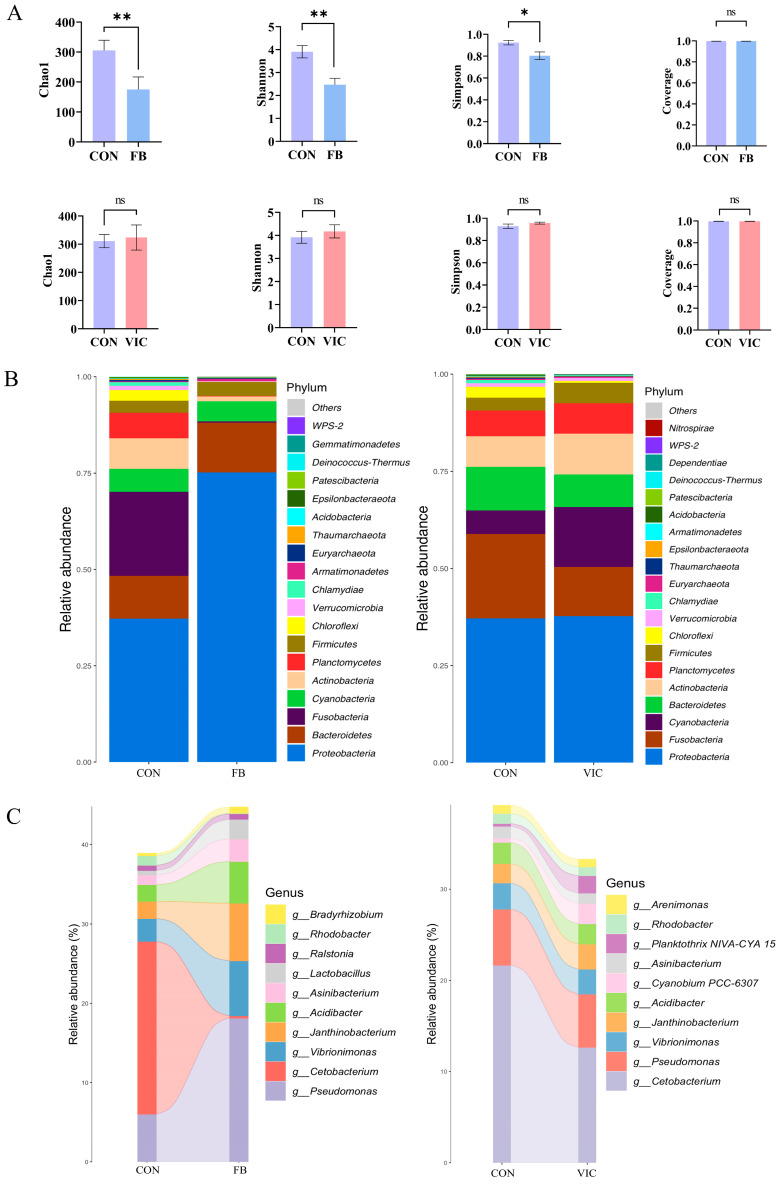
Effects of faba bean and vicine supplementation on the grass carp intestinal microbiota. (**A**) Alpha diversity indices of the intestinal microbiota (Chao1, Shannon, Simpson, Good’s coverage). (**B**) Relative abundance of intestinal microbiota at the phylum level. (**C**) Relative abundance of intestinal microbiota at the genus level. Data are presented as means ± SDs, and one-way ANOVA followed by Duncan’s test was performed to determine statistical significance, ** *p* < 0.01, or * *p* < 0.05.

As depicted in Figure 5, comparisons of intestinal microbiota diversity and composition were conducted between the control group and the faba bean group, as well as between the control group and the vicine group. Faba bean supplementation for 150 days significantly reduced alpha diversity indices (Chao1, Shannon, Simpson) in grass carp (*p* < 0.05, Figure 5A), whereas vicine supplementation had no significant effect on these parameters compared to the control.

At the phylum level (Figure 5B), *Proteobacteria* was the dominant phylum across all groups. Notably, *Proteobacteria* abundance was higher in the faba bean group than in the control group, while *Fusobacteria* and *Plancctomycetes* abundances were significantly lower in the faba bean group. In the vicine group, *Fusobacteria* and *Bacteroidetes* abundances decreased significantly compared to the control, whereas *Cyanobacteria* and *Actinobacteria* showed notable increases.

At the genus level (Figure 5C), the faba bean group exhibited the highest relative abundance of *Pseudomonas*, with significant increases in *Vibrionimonas*, *Janthinobacterium*, and *Acidibacter* and a marked decrease in *Cetobacterium*. In contrast, the vicine group displayed a significant reduction in *Cetobacterium* abundance, with no substantial changes observed in other bacterial genera.

### 3.5. Effects of Vicine on Biochemistry and Microstructure of Hepatopancreas of Grass Carp

**Figure 6:**
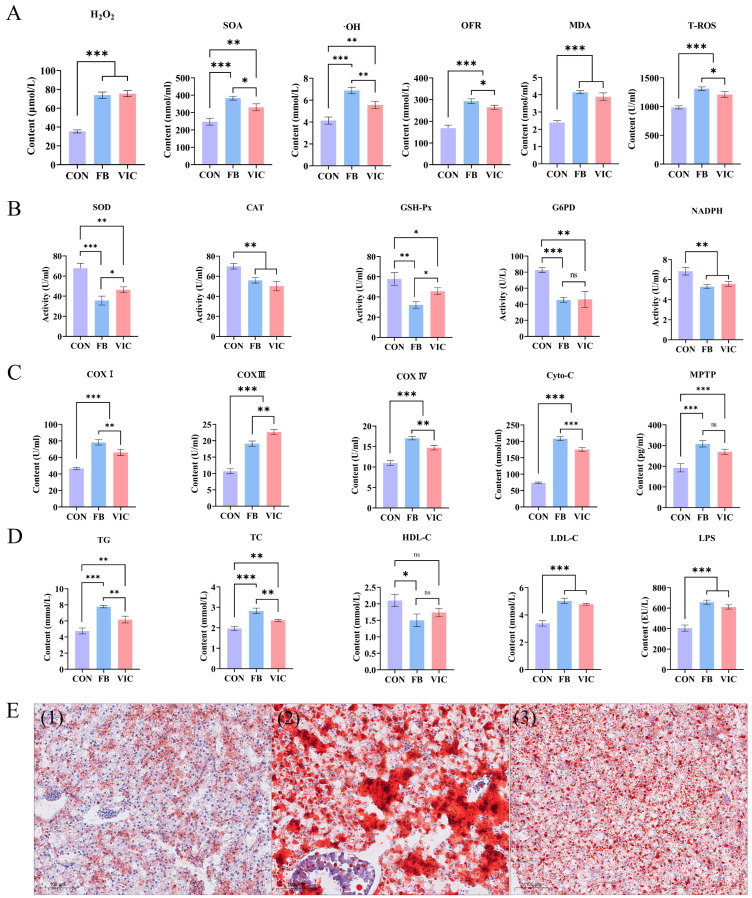
Biochemical and histological analysis of the grass carp hepatopancreas. CON: control group; FB: faba bean group; VIC: vicine group. (**A**) Quantification of ROS and oxidative products: H_2_O_2_, SOA, ·OH, OFR, MDA, and T-ROS. (**B**) Antioxidant enzyme activities and redox molecules: SOD, GSH-Px, CAT, NADPH, and G6PD. (**C**) Mitochondrial electron transport chain components: cytochrome c oxidase complexes (COXI, COXIII, COXIV), Cyto-C, and MPTP. (**D**) Lipid metabolism and inflammatory markers: TG, TC, HDL-C, LDL-C, and LPS. (**E**) Histological observation of hepatopancreas: representative micrographs of transverse sections [(1) CON, (2) FB, (3) VIC; H&E staining, scale bar = 100 μm]. For each treatment group, three ponds were used (three fish per pond), and data are presented as the mean ± SD of the ponds. Statistical significance was evaluated using one-way ANOVA followed by Duncan’s test, *** *p* < 0.001, ** *p* < 0.01, or * *p* < 0.05.

As shown in Figure 6A, ROS levels—including H_2_O_2_, SOA, ·OH, OFR, MDA, and T-ROS—were significantly higher in the hepatopancreas of grass carp in both the faba bean and vicine groups compared to the control group (*p* < 0.05). Notably, the vicine group exhibited significantly lower SOA, ·OH, OFR, and T-ROS levels than the faba bean group (*p* < 0.05), although H_2_O_2_ and MDA levels did not differ significantly between the two treatment groups.

In terms of antioxidant enzyme activities (Figure 6B), the vicine group displayed significantly higher SOD and GSH-Px activities than the faba bean group (*p* < 0.05). However, no significant differences were observed in CAT, NADPH, or G6PD between the two groups (*p* > 0.05). Importantly, all antioxidant enzyme activities in both treatment groups remained significantly lower than those in the control group (*p* < 0.05).

Mitochondrial ETC components in the grass carp hepatopancreas—including COXI, COXIII, COXIV, Cyto-C, and MPTP—were significantly elevated in both the faba bean and vicine groups compared to the control (*p* < 0.05, Figure 6C). Notably, the vicine group exhibited a distinct pattern: COXIII levels were significantly higher than those in the faba bean group, whereas COXI, COXIV, Cyto-C, and MPTP showed significantly lower levels in the vicine group compared to the faba bean group (*p* < 0.05).

Oil red O staining of the hepatopancreas (Figure 6E) revealed marked differences in lipid deposition among groups. Fat droplet area was quantified using Image-Pro Plus 6.0, with the control group serving as the reference standard. The vicine group exhibited a liver fat droplet area 1.82 ± 0.43-fold relative to the control, significantly higher than the control (*p* < 0.05). The faba bean group showed an even greater increase, with a fat droplet area of 3.52 ± 0.81-fold relative to the control (*p* < 0.05 vs. control). Notably, fat droplets in the faba bean group formed continuous sheets, indicative of severe hepatopancreatic fatty degeneration, whereas those in the vicine group remained scattered with minimal aggregation, suggesting less pronounced lipid accumulation.

### 3.6. Quantitative Real-Time PCR of Grass Carp Muscle

**Figure 7:**
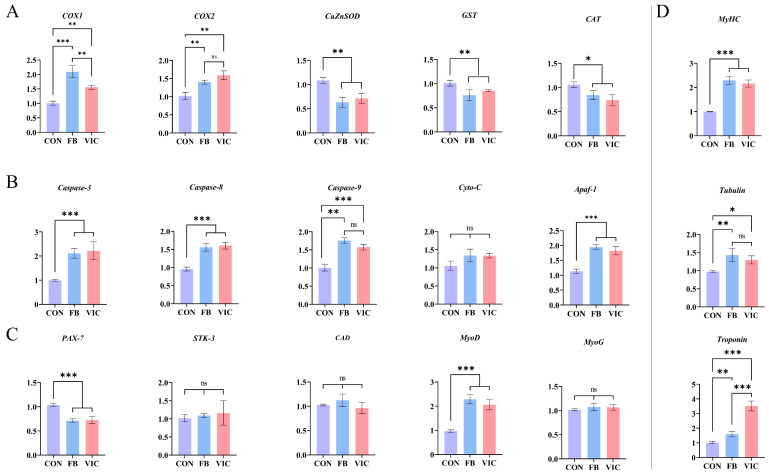
Quantitative real-time PCR of grass carp muscle. CON: control group; FB: faba bean group; VIC: vicine group. (**A**) Gene expression of ROS metabolism pathways: *COX1*, *COX2*, *CuZnSOD*, *GST*, *CAT*. (**B**) Expression of caspase-dependent apoptosis pathway genes: *Caspase-3*, *Caspase-8*, *Caspase-9*, *Cyto-C*, *Apaf-1*. (**C**) Gene expression of muscle proliferation and differentiation pathways: *Pax-7*, *STK-3*, *CAD*, *MyoD*, *MyoG*. (**D**) Expression levels of myofibrillar protein-encoding genes: *MyHC*, *Troponin*, *Tubulin*. For each treatment group, three tanks were used, with three fish per tank pooled to generate three biological replicates. Data are presented as means ± SDs, and one-way ANOVA followed by Duncan’s test was performed to determine statistical significance, *** *p* < 0.001, ** *p* < 0.01, or * *p* < 0.05.

Dietary vicine significantly up-regulated the expression of *COX1* and *COX2* in grass carp muscles (*p* < 0.05) while significantly down-regulating the expression of antioxidant enzyme-related genes (*CuZnSOD*, *GST*, and *CAT*), showing a similar trend to the dietary faba bean group (Figure 7A).

In the faba bean group, the expression of genes and factors associated with the Caspase apoptosis pathway (*Caspase-3*, *Caspase-8*, *Caspase-9* and *Apaf-1*) in grass carp muscles increased significantly. The vicine group exhibited the same up-regulation trend for these apoptotic indicators, with all differences reaching statistical significance (Figure 7B, *p* < 0.05).

In grass carp muscle, both dietary faba bean and vicine treatments significantly down-regulated the expression of *Pax-7* and up-regulated *MyoD* compared to the control (*p* < 0.05, Figure 7C). However, no significant differences were observed in the expression of other muscle proliferation/differentiation-related genes, including *STK-3*, *CAD*, and *MyoG* (*p* > 0.05).

For myofibrillar protein-encoding genes (Figure 7D), dietary faba bean and vicine significantly increased the expression of *MyHC*, *troponin*, and *tubulin* compared to the control (*p* < 0.05). Notably, the faba bean group exhibited significantly higher *troponin* expression than the vicine group (*p* < 0.05), while *MyHC* and *tubulin* levels did not differ significantly between the two treatment groups.

## 4. Discussion

Building on our previous finding that a vicine-rich faba bean aqueous extract improves grass carp muscle texture [1], the present study tested the hypothesis that the beneficial effects of feeding faba bean are attributed to vicine rather than other known anti-nutritional factors (e.g., tannins, phytic acid, and saponins) [11]. We demonstrated that inclusion of 0.6% vicine in the diet significantly improved the grass carp muscle texture, increasing both muscle fiber density and collagen content—changes that mirrored the effects observed in grass carp fed faba beans.

### 4.1. Extraction Effect of Vicine

To test our hypothesis, large-scale extraction of vicine was essential for subsequent in vivo experiments. As previously noted, research on the extraction and purification of vicine from faba beans remains limited, with significant challenges. To address these limitations, we harnessed vicine’s strong polarity to develop a cation-exchange column chromatography method for separating it from other components in faba bean water extracts. HPLC analysis revealed an initial purity of 86.4%, which was enhanced to 98.5% through recrystallization. This protocol minimizes the use of acids, alkalis, and organic reagents while requiring only basic laboratory equipment, enabling efficient vicine extraction. Compared with previously reported methods, such as trichloroacetic acid extraction [20] and prolonged organic solvent pretreatment methods [21,22], our cation-exchange column chromatography demonstrates superior extraction efficiency (97.8% vs. 60–70%) and purity (98.5% vs. 80–85%). This approach simultaneously minimizes reagent consumption and operational complexity, addressing critical limitations of traditional techniques. The developed method provides a scalable and environmentally friendly strategy for efficient vicine isolation, overcoming the inefficiencies and environmental drawbacks of conventional extraction protocols.

Notably, this study did not measure the specific content of vicine in muscle tissue; however, its safety is indirectly supported by the following evidence: ① the action of vicine relies on oxidative stress and ROS leakage induced by its intestinal metabolite (divicine), rather than direct accumulation in muscle tissue.; ② during the experimental period (150 days), the survival rate, hepatic-intestinal tissue structure, and serum bio-chemical indices of grass carp in the vicine group were significantly superior to those in the natural fava bean group (Table 2, Figure 3, Figure 4, Figure 5 and Figure 6), suggesting low risk of chronic toxicity [21]; ③ the long-term application of natural faba bean feeding in aquaculture (over 20 years) provides a practical reference for the safety of vicine [2]. Future studies may further resolve the tissue distribution characteristics of vicine and its metabolites by integrating high-sensitivity techniques such as LC-MS/MS.

### 4.2. Potential Mode of Action of Vicine in Causing Changes in Muscle Texture

As previously reported, faba beans can improve muscle texture in various aquatic animals. Meanwhile, faba bean water extract rich in vicine not only enhances grass carp muscle texture but also mitigates the adverse effects of faba beans, but the active substances that cause this effect have not yet been identified. This study’s experimental results showed that vicine, enriched in faba bean water extract, is the key active compound driving grass carp muscle texture improvement, with ROS playing a central role. Previous studies have indicated that faba bean supplementation elevates ROS levels in grass carp muscle and impairs the mitochondrial ETC, leading to excessive endogenous ROS production and reduced mitochondrial membrane potential [10]. We propose that vicine in faba beans initiates a “ROS-induced ROS-release” (RIRR) mechanism: vicine-generated ROS increases mitochondrial ETC complex activity, thereby amplifying further ROS production. However, it should be noted that both faba beans and vicine can induce substantial ROS accumulation in grass carp. Although faba bean-fed crispy grass carp has been widely consumed for decades, individuals with G6PD deficiency should still exercise caution when consuming such fish. Future studies should integrate processed food analysis and animal toxicology experiments to further validate the health impacts of vicine-fed fish consumption.

In this study, the effects of vicine on grass carp muscle were consistent with the above phenomenon, confirming that vicine was the main prooxidant of faba bean that caused changes in muscle texture (Figure 2A). Excessive ROS-induced muscle damage triggers satellite cell activation for muscle fiber repair [28]. *Pax7* and *MyoD* serve as key markers during myogenesis: *Pax7^+^/MyoD^+^* indicates satellite cell self-renewal and myofiber homeostasis, *Pax7^+^/MyoD^−^* marks activated satellite cells capable of differentiation and proliferation, while *Pax7^−^/MyoD^+^* signifies committed myoblast differentiation [29]. Our qRT-PCR analysis revealed that faba bean and vicine treatments elevated ROS levels, upregulated mitochondrial ETC complex proteins (*COX1*, *COX2*), and activated the caspase-dependent apoptotic pathway (*Caspase-3/8/9*, *Apaf-1*) in grass carp muscle (Figure 7A,B). Concurrently, we observed decreased *Pax7* expression and increased *MyoD* expression (Figure 7C), suggesting satellite cells transitioned to a differentiated (*Pax7^−^/MyoD^+^*) state. Activated caspases likely promote satellite cell differentiation into myoblasts, stimulating the generation of new muscle fibers [30,31,32], as supported by upregulated *MyHC* and *troponin* (Figure 7D).

### 4.3. Effects of Vicine on Intestinal and Hepatopancreatic Health of Grass Carp

While faba bean feeding improves grass carp muscle texture, its anti-nutritional factors (e.g., tannins, phytic acid) induce severe intestinal dysbiosis, which cascades into systemic health issues including growth retardation, fatty liver, and increased mortality (>120 days, Table 2) [11,33]. This study demonstrated that dietary vicine supplementation (0.6%) preserved the muscle-texturing benefits while alleviating intestinal damage—a key driver of faba bean toxicity.

The vicine group exhibited healthier intestinal conditions compared to the faba bean group, as reflected by the following: ① Microbial community regulation: reduced abundance of pathogenic *Pseudomonas* (Figure 5C) and increased abundance of beneficial *Cetobacterium* compared to the faba bean group, aligning with improved short-chain fatty acid synthesis and mucosal barrier function [34]; ② Structural integrity preservation: intestinal H&E staining revealed intact mucosal folds in the vicine group, in contrast to the degraded villi and thinning muscularis externa observed in the faba bean group (Figure 4D); ③ Immune function regulation: serum ACP and AKP activities—key markers of intestinal immune integrity—were significantly higher in the vicine group than in the faba bean group (Figure 3D). Although serum LPS levels did not differ significantly between groups (Figure 3D), the vicine group’s elevated ACP/AKP and preserved mucosal structure suggest enhanced resistance to endotoxin translocation. This aligns with reduced hepatic lipid accumulation (Figure 6E) and improved growth performance (higher WGR, lower FCR, Table 2). These findings indicate that purified vicine, free from antinutritional factor interference, mitigates the negative impacts of faba beans on intestinal function. This approach reduces systemic damage caused by gut microbiota imbalance and intestinal injury commonly observed with faba bean consumption [6,13,33]. Notably, however, vicine still exerts certain adverse effects on gut health. Future studies incorporating measurements of intestinal inflammatory cytokines (e.g., IL-6 and TNF-α) may further elucidate the underlying mechanisms of vicine’s impact on gut health.

Hepatopancreatic histology revealed striking differences among the groups (Figure 6E). Although aspartate (AST) and alanine (ALT) levels in the vicine group were moderately higher than those in the control group, they remained significantly lower than those in the faba bean group (Figure 3B), reflecting reduced hepatocellular damage. A parallel trend was observed in blood lipids, with faba bean feeding inducing hepatic steatosis—consistent with previous findings linking faba bean consumption to dysregulated lipid homeostasis in grass carp [35]. Notably, vicine supplementation effectively attenuated both hyperlipidemia and hepatic lipid accumulation associated with whole faba bean diets (Figure 3B and Figure 6D,E). This improvement may stem from the reduced exposure to anti-nutritional factors present in faba beans [36]. Mammalian studies suggest a link between oxidative stress, dysregulated hepatic lipid metabolism, and caspase-dependent apoptosis [37,38,39,40]. Excessive oxidative stress disrupts hepatic lipid homeostasis, thereby promoting both apoptosis and steatosis progression. In contrast, supplementation with purified vicine avoids the excessive oxidative stress and liver toxicity caused by anti-nutrients in faba beans while preserving the beneficial effects on muscle texture. The differences in liver responses demonstrated in this study highlight the value of isolating bioactive compounds like vicine.

### 4.4. Vicine May Indirectly Affect Muscle Texture Through the Gut–Muscle Axis

The intestinal microbiota plays a pivotal role in regulating fish growth, immunity, and physiological homeostasis [41]. Plant-derived bioactive compounds in feed are known to modulate microbial community structure, thereby influencing immune responses and metabolic pathways [41,42]. In this study, faba bean feeding significantly decreased the α-diversity of the intestinal microbiota and enriched *Pseudomonas* (phylum *Proteobacteria*), a pathogenic genus associated with hemorrhagic septicemia in fish [43] (Figure 5A,B). *Pseudomonas* can also secrete exotoxins to destroy the structural proteins of tissues. Additionally, faba beans increased the relative abundance of pathogenic genera *Vibrionimonas* and *Janthinobacterium*; *Janthinobacterium* is associated with systemic inflammation caused by cirrhosis [44,45]. Furthermore, faba bean feeding reduced *Cetobacterium* abundance, a genus implicated in vitamin B_12_ biosynthesis and carbohydrate metabolism [46]. Vicine supplementation did not alter microbiota α-diversity but selectively reduced *Cetobacterium* abundance (Figure 5A–C). Concurrently, it promoted the proliferation of opportunistic pathogens including *Cyanobium PCC-6307* [47] and *Planktothrix NIVA-CYA 15* [48]. These changes suggest that faba beans exert more profound detrimental effects on the grass carp intestinal microbiome than vicine, primarily through enhancing pathogenic genera and disrupting beneficial microbial functions (e.g., *Cetobacterium*-mediated metabolism). The observed microbial alterations in both groups may impose metabolic and immunological burdens on grass carp, potentially explaining the divergent growth and health outcomes between the faba bean and vicine treatments.

An alternative mechanistic framework involves the “gut–muscle axis,” through which faba beans and vicine influence the intestinal microbiota and muscle. Both treatments increased intestinal ROS and LPS levels—key substances influencing intestinal barrier dysfunction. Vicine is rapidly hydrolyzed to divicine, a potent pro-oxidant that disrupts redox homeostasis, inducing acute intestinal dysbiosis and compromising mucosal integrity. Excessive ROS not only induces intestinal microbial dysbiosis—characterized by increased *Pseudomonas* abundance and decreased *Cetobacterium* abundance—but also damages intestinal epithelial integrity, promoting the translocation of Gram-negative bacterial endotoxins (e.g., lipopolysaccharide, LPS) into the bloodstream [49]. Decreases in serum ACP, AKP, and LZM levels serve as indirect indicators of intestinal injury. LPS enters the bloodstream and translocates to hepatic tissue via the portal vein, inducing liver injury and disrupting hepatic lipid metabolism [34]. Concurrently, studies have demonstrated that LPS initiates a systemic inflammatory response through the blood circulation, which promotes muscle oxidative stress and alters muscle architecture [50]. Therefore, we believe that the intestinal ROS/LPS induced by faba beans/vicine will disrupt intestinal homeostasis and trigger a systemic inflammatory response via the blood circulation. This response impacts hepatic lipid metabolism and indirectly induces muscle tissue oxidative stress through inflammation, ultimately influencing grass carp muscle texture [49]. Thus, we conclude that LPS serves as a key mediator for both faba beans and vicine in regulating muscle structure, linking gut microbiota dysregulation to muscle ROS upregulation through the pathway of “gut microbiota dysregulation–barrier leakage–LPS translocation–oxidative stress–myofiber repair” to influence muscle texture. However, in this study, both faba beans and vicine significantly impacted the gut microbiota, and the intestinal safety of vicine still requires further mechanistic investigation for validation.

## 5. Conclusions

This study identified vicine as the key bioactive compound in faba beans for improving grass carp muscle texture, acting through the modulation of multi-organ oxidative stress and gut–muscle axis regulation. Mechanistically, vicine induces myofiber regeneration via a Reactive Oxygen-Induced Release (RIRR) mechanism, activating Caspase-3-mediated muscle satellite cell differentiation. Concomitantly, vicine-driven changes in intestinal microbiota elevate LPS, which indirectly amplifies muscle ROS levels through the gut–muscle axis. Although vicine induces oxidative stress and mild gut dysbiosis, it exhibits significantly fewer adverse effects on growth performance and intestinal histology than faba beans. These findings confirm that vicine represents a purified alternative to faba beans, offering new strategies for muscle quality improvement in crispy grass carp and other aquatic species. Further studies on dose optimization and tissue deposition are warranted to guide its practical application in aquaculture.

## Figures and Tables

**Table 1:** The composition of experimental diets: ingredients and nutritional composition (on a dry matter basis).

Ingredients	Control	Faba Bean	Vicine
Fish meal/%	3.00	0.30	3.00
Chicken meal/%	3.00	0.30	3.00
Soybean meal/%	25.00	2.50	25.00
Rapeseed meal/%	22.00	2.20	22.00
Rice bran/%	3.00	0.30	3.00
Wheat flour/%	36.00	3.60	36.00
Soybean oil/%	3.00	0.30	3.00
Ca(H_2_PO_4_)_2_/%	2.00	0.20	2.00
Bentonite/%	2.00	0.20	1.40
Premix */%	1.00	0.10	1.00
Faba bean/%	0.00	90.00	0.00
Vicine/%	0.00	0.00	0.60
Total/%	100.00	100.00	100.00
Crude protein/%	29.31	30.07	29.42
Crude fat/%	4.92	4.77	4.89
Calcium/%	0.67	0.76	0.67
Moisture/%	10.90	11.50	11.02
Crude fiber/g/kg	28.00	30.00	28.00

Notes: Control: common diet group; Faba bean: Faba bean group (simulating traditional crisp grass carp farming); Vicine: Vicine diet group. To ensure nutrient consistency, the faba bean group was formulated to match the control’s crude protein (29–30%), crude fat (4.7–4.9%), and energy levels by adjusting the non-faba bean components. Premix *: iron 5 g/kg, copper 100 mg/kg, zinc 1.5 g/kg, magnesium 20 g/kg, iodine 15 mg/kg, cobalt 5 mg/kg, selenium 5 mg/kg, manganese 0.5 g/kg, vitamin A 180,000 IU/kg, vitamin B1 0.2 g/kg, vitamin B2 0.25 g/kg, vitamin B6 0.2 g/kg, vitamin B12 0.65 mg/kg, vitamin C 2.5 g/kg, vitamin D 340,000 IU/kg, vitamin E 1.6 g/kg, vitamin K 30.05 g/kg, niacin 0.65 g/kg, calcium pantothenate 0.65 g/kg, folic acid 0.03 g/kg, inositol 1 g/kg, biotin H 8.3 mg/kg. The bentonite content in the vicine group was reduced to 14 g/kg to accommodate the 6 g/kg vicine addition, while maintaining the same total formula weight and nutrient balance.

**Table 2:** Growth parameters and muscle fiber diameter and density parameters of grass carp.

Items	Control	Faba Bean	Vicine
TBW (g)	525.90 ± 37.10 ^a^	334.44 ± 12.36 ^c^	467.74 ± 9.75 ^b^
WGR (%)	192.17 ± 16.72 ^a^	85.80 ± 6.86 ^c^	159.85 ± 5.63 ^b^
CF (g/cm^3^)	2.21 ± 0.22 ^a^	1.52 ± 0.03 ^b^	2.22 ± 0.15 ^a^
VSI (%)	5.93 ± 0.61 ^b^	8.19 ± 0.88 ^a^	7.71 ± 0.58 ^a^
HSI (%)	1.73 ± 0.09 ^b^	1.94 ± 0.22 ^a^	1.98 ± 0.10 ^a^
AFI (%)	3.27 ± 0.15 ^c^	4.44 ± 0.38 ^b^	5.15 ± 0.19 ^a^
FCR (%)	2.90 ± 0.25 ^c^	4.67 ± 0.27 ^a^	3.47 ± 0.12 ^b^
SR (%)	100 ^a^	88.00 ± 2.66 ^a^	100 ^a^

Notes: Control: common diet group; Faba bean: Faba bean group; Vicine: Vicine diet group; WGR: Weight gain rate; CF: Condition factor; VIS: Visceral somatic index; HIS: Hepatopancreas somatic index; AFI: Abdominal fat index; FCR: Feed conversion rate; SR: Survival rate. For each treatment group, n = 3 ponds were used, with 3 fish per pond. Data are presented as the mean ± SD of the ponds. Different letters in the same line represent a significant difference *p* < 0.05.

## Data Availability

Data is contained within the article and Supplementary Material.

## Supplementary Materials

The following supporting information can be downloaded at: https://www.mdpi.com/article/10.3390/antiox14070813/s1, Figure S1: Vicine content in faba beans from different origins; Table S1: Summary of statistical parameters (F values and exact *p*-values) for all analyses.
