# Supplementary figures and images for: The Naturally Bioactive Vicine Extracted from Faba Beans Is Responsible for the Transformation of Grass Carp (Ctenopharyngodon idella) into Crisp Grass Carp
Source: Antioxidants (Basel). 2025 Jul 1;14(7):813. doi: 10.3390/antiox14070813 (PMC12291655; doi:10.3390/antiox14070813)

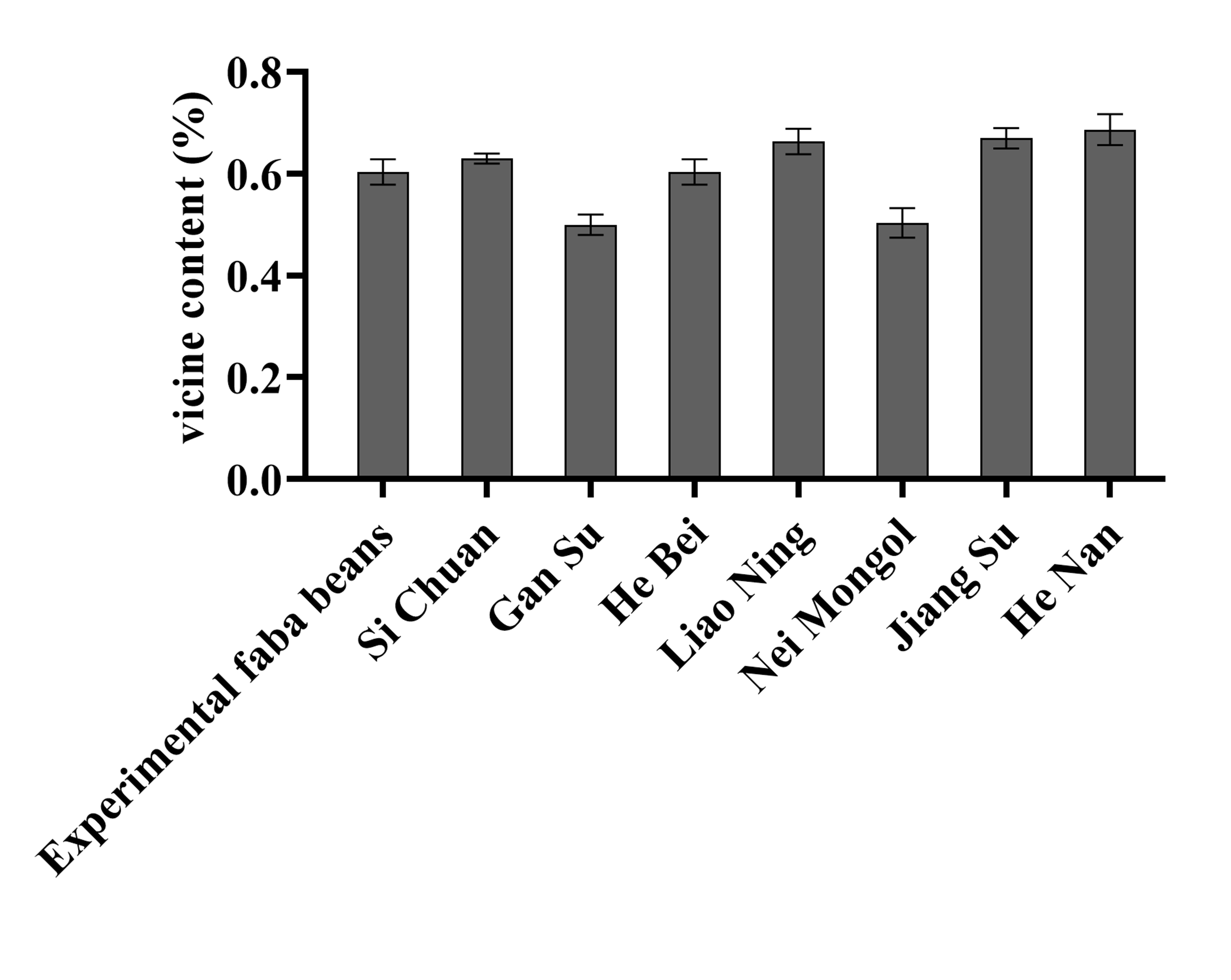

Supplement: Supplementary file 1 [file antioxidants-14-00813-s001.zip › Figure S1. Vicine content in faba beans from different origins.jpg]
